# Small-Molecule-Based Lineage Reprogramming Creates Functional Astrocytes

**DOI:** 10.1016/j.celrep.2016.06.042

**Published:** 2016-07-07

**Authors:** E Tian, Guoqiang Sun, Guihua Sun, Jianfei Chao, Peng Ye, Charles Warden, Arthur D. Riggs, Yanhong Shi

**Affiliations:** 1 Division of Stem Cell Biology Research, Department of Developmental and Stem Cell Biology, Beckman Research Institute of City of Hope, 1500 E. Duarte Road, Duarte, CA 91010, USA; 2 Diabetes and Metabolism Research Institute, City of Hope, 1500 E. Duarte Road, Duarte, CA 91010, USA; 3 Integrative Genomics Core, Beckman Research Institute of City of Hope, 1500 E. Duarte Road, Duarte, CA 91010, USA

## Abstract

Growing evidence indicates important roles for astrocytes in neurodevelopment and diseases. However, astrocytes and their roles in these processes remain poorly understood. Despite recent progress in reprogramming somatic cells into different types of neural cells, reprogramming to astrocytes has lagged. Here, we show that functional astrocytes can be generated from mammalian fibroblasts using only small molecules. Induced mouse astrocytes resemble primary astrocytes in astrocytic gene expression and epigenomic status and exhibit functional properties in promoting neuronal maturation, glutamate uptake, and calcium signaling. Moreover, these cells can recapitulate the Alexander disease phenotype of protein aggregation when expressing Gfap with a disease-causing mutation. The same compounds can also reprogram human fibroblasts into astroglial progenitor cells that can further mature into functional astrocytes. These chemically induced astrocytes may provide cellular models to uncover roles of astrocytes in normal neurodevelopment and pathogenesis of neurological diseases.

## In Brief

In this study, Tian et al. report that a small-molecule cocktail can directly convert mouse and human fibroblasts into mature, functional astrocytes without the use of transgenes. These chemically induced astrocytes resemble primary astrocytes in gene expression and function and can be used to study diseases of astrocyte dysfunction.

## Graphical Abstract

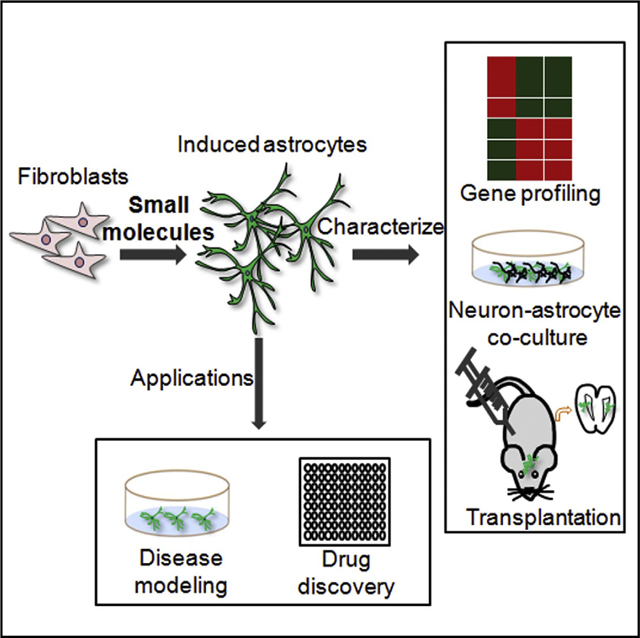

## INTRODUCTION

Astrocytes are glial cells that are located in all regions of the brain (Molofsky et al., 2012; Verkhratsky et al., 2012). They have long been held as the supporting components in neural tissues (Wang and Bordey, 2008; Sofroniew and Vinters, 2010). However, over the past decades, increasing evidence has established a variety of essential functions for astrocytes in neural development and in the pathogenesis of neurological diseases (Verkhratsky et al., 2012). Astrocytes play a critical role in neuronal maturation, synapse formation and plasticity, and glutamate clearance to reduce excitotoxicity (Banker, 1980; Song et al., 2002; Hama et al., 2004; Eroglu and Barres, 2010). Astrocyte dysfunction contributes to many neurodegenerative diseases and is the direct cause for some neurological disorders (Molofsky et al., 2012; Verkhratsky et al., 2012), such as Alexander disease (AxD) (Messing et al., 2012). Despite increasing data revealing new roles for astrocytes, our knowledge on astrocytes remains largely behind what we know about their neuronal counterpart. There is an urgent need to establish new cellular models for astrocytes to uncover their versatile roles in the nervous system.

Expression of lineage-specific factors has been shown to induce cell fate change, including reprogramming somatic cells to induced pluripotent stem cells (iPSCs) (Takahashi and Yamanaka, 2006) and converting one type of somatic cells to another (Davis et al., 1987). The latter is also called direct reprogramming or conversion. Extensive efforts have been devoted into converting somatic cells, like fibroblasts, into different types of neural cells, such as neural stem cells (Kim et al., 2011; Han et al., 2012; Lujan et al., 2012), neurons (Vierbuchen et al., 2010; Caiazzo et al., 2011; Pang et al., 2011; Yoo et al., 2011), and oligodendrocytes (Najm et al., 2013; Yang et al., 2013). However, direct reprogramming of somatic cells into astrocytes has just begun (Caiazzo et al., 2015).

Introducing exogenous factors in reprogramming has raised various concerns, including the risk of insertional mutagenesis and genetic alteration associated with retroviral delivery (Hawley, 2008) and low reprogramming efficiency associated with episomal transfection (Okita et al., 2008). During the course of this study, cocktails of small molecules were shown to convert mouse or human fibroblasts into neurons (Hu et al., 2015; Li et al., 2015). However, no chemical reprogramming has been reported to change fibroblasts, or any other mature cell types, to astrocytes yet. Here, we demonstrate that small molecules can be used to directly convert fibroblasts into functional astrocytes without transgenes.

## RESULTS

### A Compound Cocktail Induces the Conversion from MEFs to Astrocyte-like Cells

During our search for small molecules that can reprogram somatic cells into iPSCs, a chemical cocktail VC6TFZ was used to reprogram mouse embryonic fibroblasts (MEFs) into iPSCs (Hou et al., 2013). This compound combination includes the histone deacetylase inhibitor VPA (V), the GSK3β inhibitor compounds can reprogram MEFs into GFAP-positive and S100β-positive astrocyte-like cells.

### A TGFβ Inhibitor Is Critical for Astrocytic Conversion

Next, we sought to identify compounds critical for astrocytic conversion. The combination of VC6, V6, or 6 alone was CHIR99021 (C), the TGFβ inhibitor 616452 (6), the lysine specific histone demethylase LSD1 inhibitor tranylcypromine (T), the cyclic AMP inducer forskolin (F), and a histone methylation inhibitor DZNep (Z). In this chemical cocktail, compounds F and Z were used together to induce the expression of Oct4, a factor critical for reprogramming. We have previously identified the small molecule OAC1 as an Oct4-activating compound (Li et al., 2012). In this study, we tested whether the combination of VC6T with the OAC1 compound (together termed VC6TO) could reprogram MEFs into iPSCs. MEFs were derived from mice harboring an Oct4 promoter-driven GFP (OG2) reporter. Treatment with VC6TO for up to 25 days failed to induce any Oct4-GFP-positive iPSC colonies from the OG2 MEFs. Instead, we observed cells with astrocyte-like morphology (Figure S1A).

**Figure 1.**
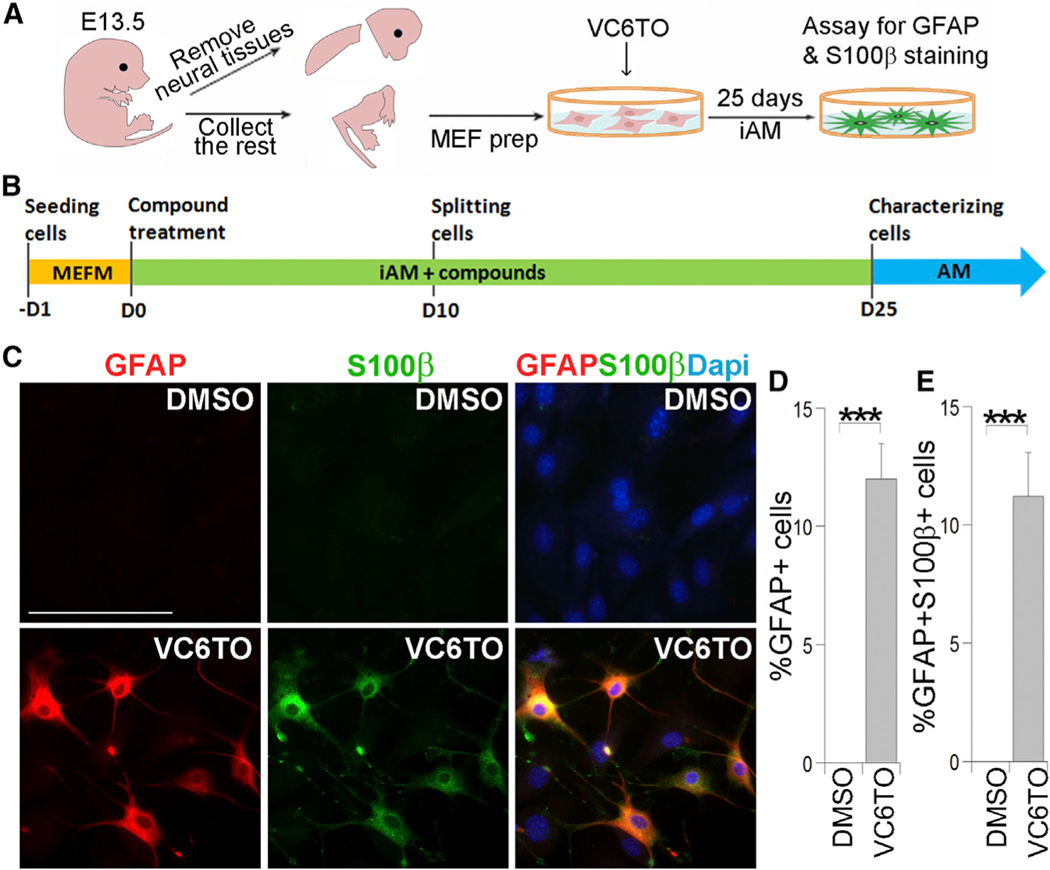
Direct Reprogramming MEF into Astrocyte-like Cells by VC6TO (A) The scheme of MEF preparation is shown. (B) The scheme of astrocytic reprogramming with compound treatment. AM, astrocyte medium; iAM, induced astrocyte medium; MEFM, MEF medium. (C) Immunostaining for GFAP and S100β in cells derived from MEFs treated with DMSO or VC6TO for 25 days. Nuclei were counter-stained with DAPI. The scale bar represents 100 μm. (D and E) The percentage of GFAP-positive cells and GFAP and S100β double-positive cells from total cells is shown. n = 3,000–4,000 cells. See also Figure S1. For all quantifications in Figures 1, 2, 3, 4, 5, 6, and 7, error bars are SD of the mean; *p < 0.05; **p < 0.01; ***p < 0.001.

**Figure 2.**
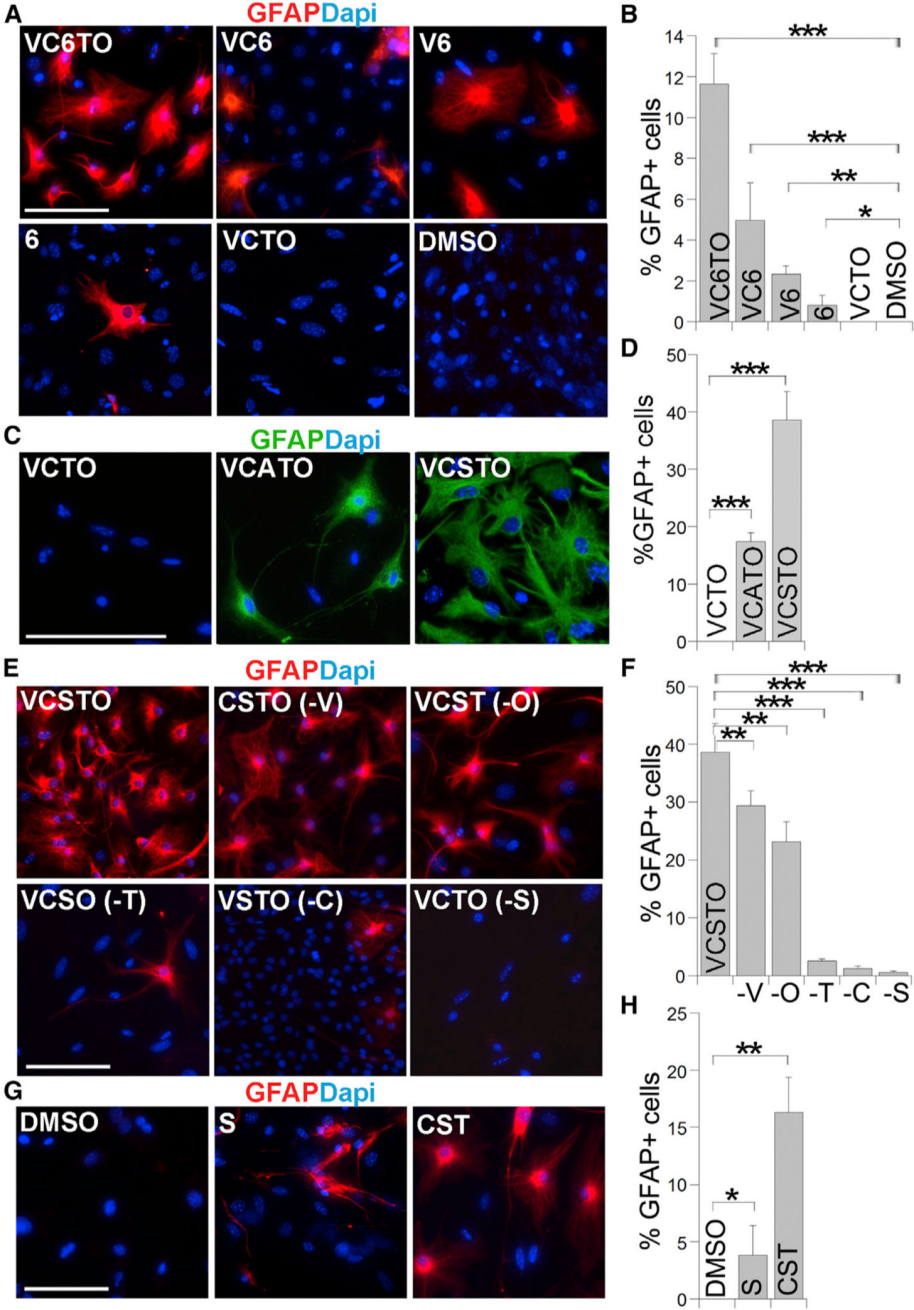
A TGFβ Inhibitor Is Critical for Astrocytic Reprogramming (A, C, E, and G) Immunostaining for GFAP in cells derived from MEFs treated with DMSO control or different compound combinations for 25 days. Nuclei were counter-stained with DAPI. The scale bar represents 100 μm. (B, D, F, and H) The percentage of GFAP-positive cells in MEFs treated with individual compound combinations described in (A), (C), (E), and (G). n = 2,000–6,000 cells. See also Figures S2 and S3.

This observation triggered us to test whether the VC6TO cocktail could reprogram MEFs into astrocytes. We paid special attention to exclude any neural tissues from the MEF preparation (Figure 1A) as described (Vierbuchen et al., 2010). Immunostaining MEFs with various neural lineage markers revealed no contamination of neural progenitor cells, neurons, astrocytes, and oligodendrocyte progenitor cells (Figure S1B); instead, 99.6% of cells expressed the fibroblast marker FSP1 (Figure S1C). These MEFs were treated with VC6TO and cufltured in induced astrocyte medium (iAM) (Figure 1B). Twenty-five days after compound treatment, we immunostained the resultant cells for astrocyte markers, glial fibrillary acidic protein (GFAP) and S100β. We detected 12% GFAP-positive cells with typical astrocyte morphology (Figures 1C and 1D). The percentage of GFAP+S100β+ cells was similar to that of GFAP+ cells (Figure 1E). Together, these results indicate that the VC6TO able to induce GFAP-positive cells from MEFs, although the efficiency of conversion decreased when the number of compounds was reduced. In contrast, subtraction of compound 6 from VC6TO led to failure of astrocytic conversion, as revealed by the lack of GFAP-positive cells (Figures 2A and 2B). These results suggest that compound 6 is necessary and sufficient to induce the conversion of MEFs into astrocyte-like cells.

Because compound 6 is a transforming growth factor β (TGFβ) receptor 1 kinase inhibitor (Gellibert et al., 2004), we asked whether other inhibitors of TGFβ receptor 1 could induce astrocytic reprogramming together with VCTO. We tested A-83–01 (A) or SB-431542 (S), two well-characterized inhibitors of TGFβ receptor 1 (Inman et al., 2002; Tojo et al., 2005). Treating MEFs with either the combination of VCTO with A (VCATO) or VCTO with S (VCSTO) induced a substantial increase in GFAP-positive cells with astrocyte morphology (Figures 2C and 2D). In contrast, VCTO did not induce any GFAP-positive cells (Figures 2C and 2D). We noticed that VCATO and VCSTO induced more GFAP-positive cells than the initial VC6TO combination (Figures 2B–2D), with the highest conversion efficiency observed with VCSTO, which induced 38% GFAP-positive cells at day 25 after compound treatment. We therefore focused the rest of the study on VCSTO-induced reprogramming.

We subtracted individual compounds from VCSTO to determine the effect of individual compounds on astrocytic conversion. Similar to removal of 6, subtraction of S from VCSTO led to almost complete loss of GFAP-positive cells (Figures 2E and 2F). Subtraction of T or C decreased reprogramming efficiency dramatically, whereas removal of V or O reduced the efficiency mildly (Figures 2E and 2F). On the other hand, compound S by itself was sufficient to induce GFAP-positive cells from MEFs (Figures 2G and 2H). Combination with C and T led to a more-robust induction of GFAP-positive cells (Figures 2G and 2H). These results indicate that compound S is critical for astrocytic reprogramming, whereas compounds C and T promote reprogramming efficiency.

Having identified S as a critical compound for astrocytic reprogramming, next we determined the dose response of compound S. We treated MEFs with VCSTO at different concentrations of S from 0 to 10 mM and observed increased GFAP-positive cells with elevated concentrations of S (Figures S2A and S2B). No toxicity was observed in cells treated with VCSTO at the concentration of S at 10 μM or even higher (20 or 30 μM; Figure S2C). Because S is an inhibitor of TGFβ receptor 1 (Inman et al., 2002), we asked whether the TGFβ signaling is suppressed by VCSTO treatment. After 24-hr VCSTO treatment of MEFs, dramatic inhibition of gene expression was observed for a set of TGFβ downstream effectors (Figure S2D), including Atf4, Col1a1, decorin (Dcn), Gadd45b, Ifrd1, p21, p27, Tgfb1i1, Tgfbi, and Tsc22d1, consistent with the role of S in inhibiting TGFβ signaling. These results indicate that a TGFβ inhibitor is critical for converting fibroblasts into astrocyte-like cells.

To determine whether astrocyte-like cells could also be converted from other cell types, we treated mouse tail-tip fibro-blasts (TTFs) with VCSTO compounds. Twenty-five days after VCSTO treatment, GFAP-positive and S100b-positive cells with astrocyte morphology were detected (Figure S3A). The percentage of GFAP+S100β+ cells was similar to that of GFAP+ cells (Figures S3A–S3C).

### VCSTO-Induced Cells Express Astrocytic Genes and Exhibit Epigenetic Reprogramming

**Figure 3.**
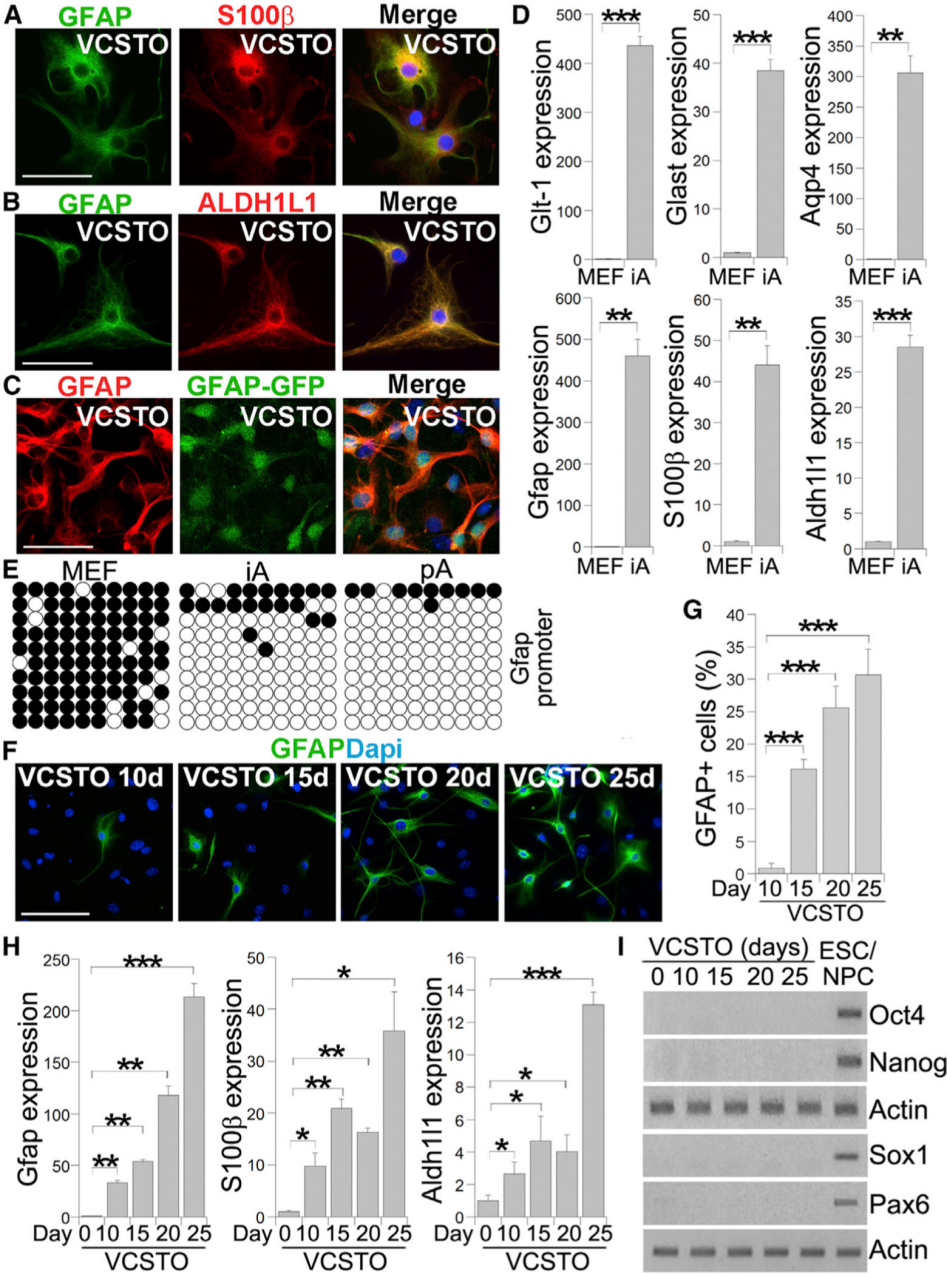
Expression of Astrocytic Markers from VCSTO-Induced Cells (A–C) Immunostaining for GFAP and S100β (A), GFAP and ALDH1L1 (B), and GFAP and visualizing GFAP-GFP (C) in VCSTO-induced cells. Nuclei DAPI staining (blue) is included in the merged images. The scale bar represents 50 μm. In (C), MEFs from GFAP-GFP mice were induced by VCSTO. (D) The expression of astrocyte-related genes in VCSTO-iAs, relative to MEF, measured by realtime PCR. The expression in MEF was defined as 1. n = 3 experimental repeats. (E) Bisulfite sequencing the Gfap promoter regionin MEFs, iAs, and mouse pAs. Open and closed circles indicate unmethylated and methylated CpGs, respectively. (F) Immunostaining for GFAP in cells treated with VCSTO for 10, 15, 20, and 25 days. The scale bar represents 100 mm. (G) The percentage of GFAP-positive cells in cells treated with VCSTO for different days. (H) Real-time PCR of astrocyte markers at different days of VCSTO treatment. The expression at day 0 was defined as 1. n = 3 experimental repeats. (I) RT-PCR of pluripotency markers, Oct4 and Nanog, and neural progenitor markers, Sox1 and Pax6, during the time course of VCSTO treatment. RNA from mouse embryonic stem cells (ESCs) and neural progenitor cells (NPCs) was included as positive controls.

To verify that the VCSTO-reprogrammed cells were indeed astrocytes, we first determined astrocytic marker expression in these cells. Double staining for GFAP and S100β revealed that the VCSTO-induced cells expressed both GFAP and S100β (Figure 3A). In addition to GFAP and S100β, ALDH1L1 has been identified to be a reliable marker for astrocytes (Barres, 2008). Double staining the VCSTO-reprogrammed cells with GFAP and ALDH1L1 revealed that the compound-induced cells were positive for both of these astrocytic markers (Figure 3B). These results further confirmed the astrocyte identity of the VCSTO-induced cells.

Because astrocytes could be visualized by GFP fluorescence in the GFAP-GFP reporter mice (Zhuo et al., 1997), we derived MEFs from these mice and treated them with VCSTO to monitor astrocytic conversion. GFAP-GFP-positive cells emerged around days 10–15 after VCSTO treatment. The induced cells were visualized for GFAP-GFP fluorescence and immunostained for GFAP at day 25 after VCSTO treatment. Nearly all GFAP-GFP positive cells were also positive for GFAP immunostaining (Figure 3C).

Astrocytes express high levels of glutamate transporters, predominantly GLT-1 and GLAST (Chaudhry et al., 1995). Aquaporin 4 (AQP4), a member of the aquaporin family of membrane proteins, is also enriched in astrocytes (Simard and Nedergaard, 2004). Real-time PCR revealed that the VCSTO-reprogrammed cells expressed high levels of Glt-1, Glast, and Aqp4, in addition to the astrocytic markers Gfap, S100β, and Aldh1l1 (Figure 3D), further strengthening our conclusion that the VCSTO-reprogrammed cells are astrocytes.

An important aspect of reprogramming is epigenetic reprogramming. Demethylation of the *Gfap* promoter has been shown to be associated with astrocyte differentiation (Hatada et al., .,2008). We tested whether the *Gfap* promoter is demethylated during astrocytic conversion from MEFs. VCSTO-induced cells were sorted for GFAP-positive cells after GFAP staining. The resultant cells were subjected to DNA methylation analysis. Bisulfite sequencing revealed that the *Gfap* promoter of VCSTO-induced cells was largely demethylated, similar to that in primary astrocytes (pAs) (Figure 3E), whereas the *Gfap* pro-moter in parental MEFs was highly methylated (Figure 3E). This result indicates that epigenetic reprogramming occurred during VCSTO-induced astrocytic conversion.

**Figure 4.**
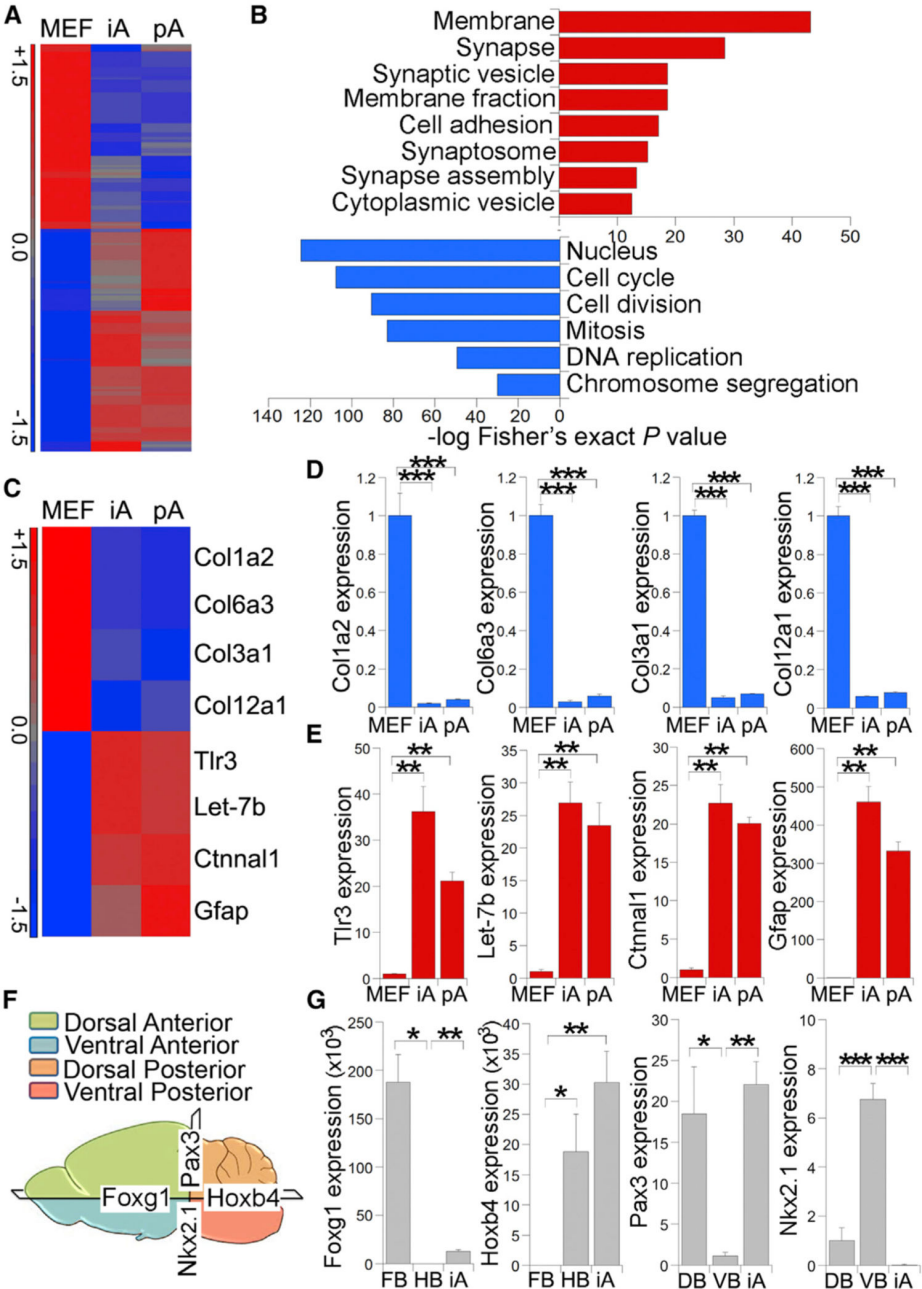
Genomic-wide Transcriptional Profiling of VCSTO-iAs (A) Heatmap presentation of microarray analysis of MEFs, VCSTO-iAs, and mouse pAs. Genes upregulated in iAs and pAs, compared to MEFs, are shown in red, whereas genes downregulated in iAs and pAs, compared to MEFs, are shown in blue. (B) GO terms for genes upregulated in both iAs and pAs, relative to MEFs, are shown in red, whereas GO terms associated with genes downregulated in iAs and pAs are shown in blue. The x axis represents enrichment scores, with p value calculated via Fisher’s exact test. (C) Heatmap presentation of a selected set of fibroblast-related genes (the upper four genes) and astrocyte-related genes (the lower four genes). (D and E) Real-time PCR validation of the expression of four fibroblast-associated genes (D) and four astrocyte-associated genes (E). The expression in MEFs was defined as 1. n = 3 experimental repeats. (F) Schematic presentation of different regions of the brain. (G)Relative expression of regional subtype markers in iAs measured by real-time PCR. DB, dorsal brain; FB, forebrain; HB, hindbrain; VB, ventral brain. n = 3 experimental repeats. See also Figure S4.

To determine the dynamic expression pattern of astrocytic genes during the conversion, we treated MEFs with VCSTO for various time periods and performed immunostaining and RT-PCR at days 10, 15, 20, and 25. We could see GFAP-positive cells at day 10 after compound treatment, although the efficiency was low (Figures 3F and 3G). By day 15, we detected more than 15% GFAP-positive cells. The rate of conversion further increased with time. By day 25, GFAP-positive cells reached more than 30% (Figures 3F and 3G). In parallel RT-PCR analysis, we observed increased expression of the astrocytic markers Gfap, S100b, and Aldh1l1 along the time course, with the highest induction at day 25 (Figure 3H). In contrast, we did not observe the induction of the pluripotency genes Oct4 and Nanog, and the neural progenitor genes Sox1 and Pax6, during the same characteristic of MEFs was globally reprogrammed toward that of astrocytic lineage (Figure 4A). Hierarchical clustering revealed that the overall gene expression pattern in induced astrocytes (iAs) is more similar to that in pAs than to parent MEFs (Figures S4A and S4B). Among the genes upregulated (≥2-fold) in pAs compared to MEFs, 53.9% were also upregulated in iAs; among the genes downregulated in pAs relative to MEFs, 68.3% were also downregulated in iAs. Genes upregulated in both iAs and pAs, compared to MEFs, were significantly enriched for gene ontology (GO) terms associated with membrane and synapse (Figure 4B), consistent with the critical time course (Figure 3I), suggesting that these compounds induce astrocytic conversion without inducing iPSC or neural progenitor cell intermediates.

### Genome-wide Remodeling and Regional Specificationin VCSTO-Induced Astrocytes

Next, we performed genome-wide profiling to compare gene expression pattern of VCSTO-induced astrocytes with that of pAs and MEFs. MEFs were derived from GFAP-GFP reporter mice and treated with VCSTO for 25 days. The reprogrammed cells were sorted for GFAP-GFP-positive cells and subjected to DNA microarray analysis, along with pAs and MEFs. A heatmap depicting all probe sets that were differentially expressed by at least 1.5-fold showed that the transcriptional program role of astrocytes in synaptogenesis (Hama et al., 2004; Eroglu and Barres, 2010). In contrast, genes downregulated in both iAs and pAs, compared to MEFs, were significantly enriched for GO terms linked to cell cycle and cell division (Figure 4B).

Validation of differentially expressed genes revealed that the known fibroblast-related genes were downregulated in both VCSTO-iAs and pAs, compared to MEFs (Figures 4C, 4D, and S4C). In contrast, genes that are known to be expressed in astrocytes or involved in astrocyte differentiation and functions were strongly upregulated in both iAs and pAs, compared to MEFs (Figures 4C, 4E, and S4D). These results indicate that iAs resemble pAs in genome-wide gene expression profile.

To determine the regional subtypes of VCSTO-iAs, we performed real-time PCR to measure the expression levels of barely detectable Nkx2.1 and Lix1 expression (Figures 4G and S4E). These results indicate that the chemical iAs can be regionally specified.

### VCSTO-iAs Are Functional

**Figure 5.**
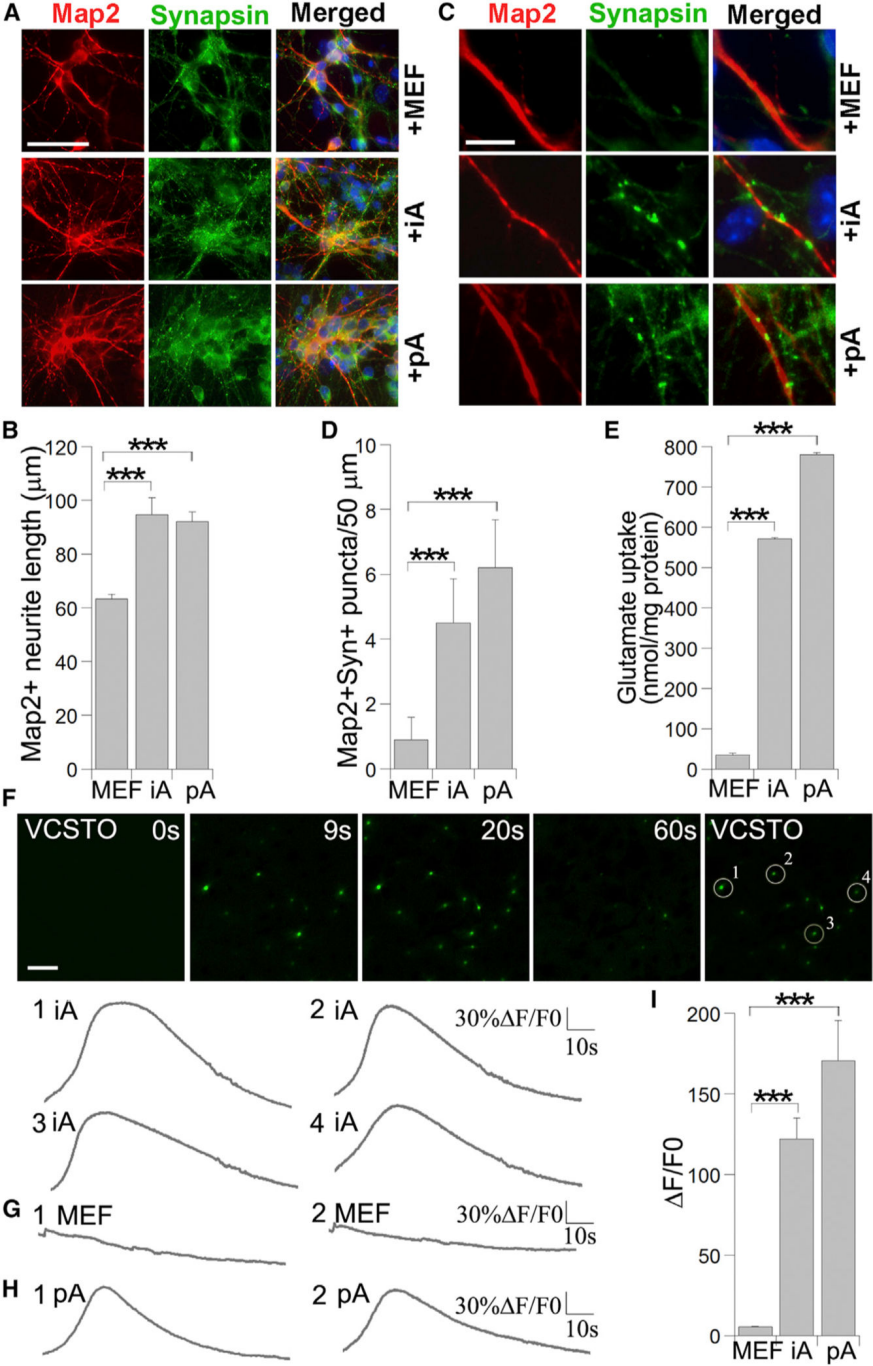
Compound-iAs Are Functional (A) Immunostaining mouse primary cortical neurons for Map2 and synapsin after co-culturing with MEF, iAs, and mouse pAs for 5 days. The scale bar represents 50 μm. (B) Quantification of Map2+ neurite length in neurons co-cultured with MEFs, iAs, and pAs. n = 1,000 cells. (C) Increased Map2+synapsin+ puncta in neuronsco-cultured with iAs and pAs, relative to co-culture with MEFs. The scale bar represents 10 μm. (D) Quantification of Map2+synapsin+ puncta per 50-mm neurite length in neurons co-cultured with MEFs, iAs, and pAs. n = 30 neurites. (E) Measurement of glutamate uptake in iAs, pAs, and MEFs. n = 3 experimental repeats. (F) Calcium signal change in response to glutamate stimulation is shown by calcium reporter fluorescent dye intensity change (ΔF/F_0_) over time (seconds) in iAs. The scale bar represents 100 mm. (G) Lack of calcium spikes after glutamate stimulation in MEFs. (H) Calcium spikes after glutamate stimulation in pAs. (I) Quantification of ΔF/F_0_ in MEFs, iAs, and pAs in response to glutamate stimulation. n = 400–600 cells. See also Figure S5.

To test whether VCSTO-iAs possess astrocyte function to promote neuronal maturation and synaptic formation, we co-cultured them with mouse primary cortical neurons. Neuronal maturation was evaluated by immunostaining with a mature neuronal marker, MAP2, at day 5 after co-culture. Both total neurite length and neurite complexity were increased in neurons co-cultured with iAs or pAs, compared to that in neurons co-cultured with MEFs (Figures 5A, 5B, and S5). Moreover, the density of synap-sin-positive puncta along the MAP2-positive neurites was significantly increased markers for forebrain (Foxg1, Otx1, and Otx2), hindbrain (Hoxb4, Egr2, and Grx2), dorsal (Pax3 and Trhr) and ventral (Nkx2.1 and Lix1) brains (Figures 4F and S4E). iAs expressed both the forebrain markers Foxg1, Otx1, and Otx2 and the hindbrain markers Hoxb4, Egr2, and Grx2, although the expression of the hindbrain markers is more robust (Figures 4G and S4E), suggesting that iAs contain both anterior and posterior astrocyte subtypes, perhaps with a more-abundant subpopulation of posterior astrocytes. As for the dorsal-ventral regionality, VCSTO-iAs are predominantly dorsal, exhibiting robust Pax3 and Trhr expression but in neurons co-cultured with iAs and pAs, compared to that in neurons co-cultured with MEFs (Figures 5C and 5D). These results indicate that the VCSTO-iAs exhibit functional property in promoting neuronal maturation and synaptogenesis, like pAs.

Next, we determined whether the compound-iAs were functional in glutamate uptake. pAs, iAs, and parental MEFs were cultured in media containing glutamate for 6 hr; the concentration of glutamate in the media was measured to determine glutamate uptake. Both iAs and pAs exhibited substantial glutamate uptake, compared to MEFs (Figure 5E), indicating that compound-iAs are functional in glutamate uptake.

Calcium imaging analysis revealed that the VCSTO-iAs exhibited glutamate-induced calcium spikes, in a manner similar to pAs, whereas MEFs did not respond to glutamate stimulation with calcium spikes (Figures 5F–5I). These results suggest that iAs acquire the ability to respond to neurotransmitters through calcium signaling, like pAs. In summary, the chemical iAs are functional astrocytes with the ability to promote neuronal survival and maturation, uptake glutamate, and respond to calcium signaling.

### VCSTO-iAs Can Survive and Retain Astrocyte Identity In Vivo

**Figure 6.**
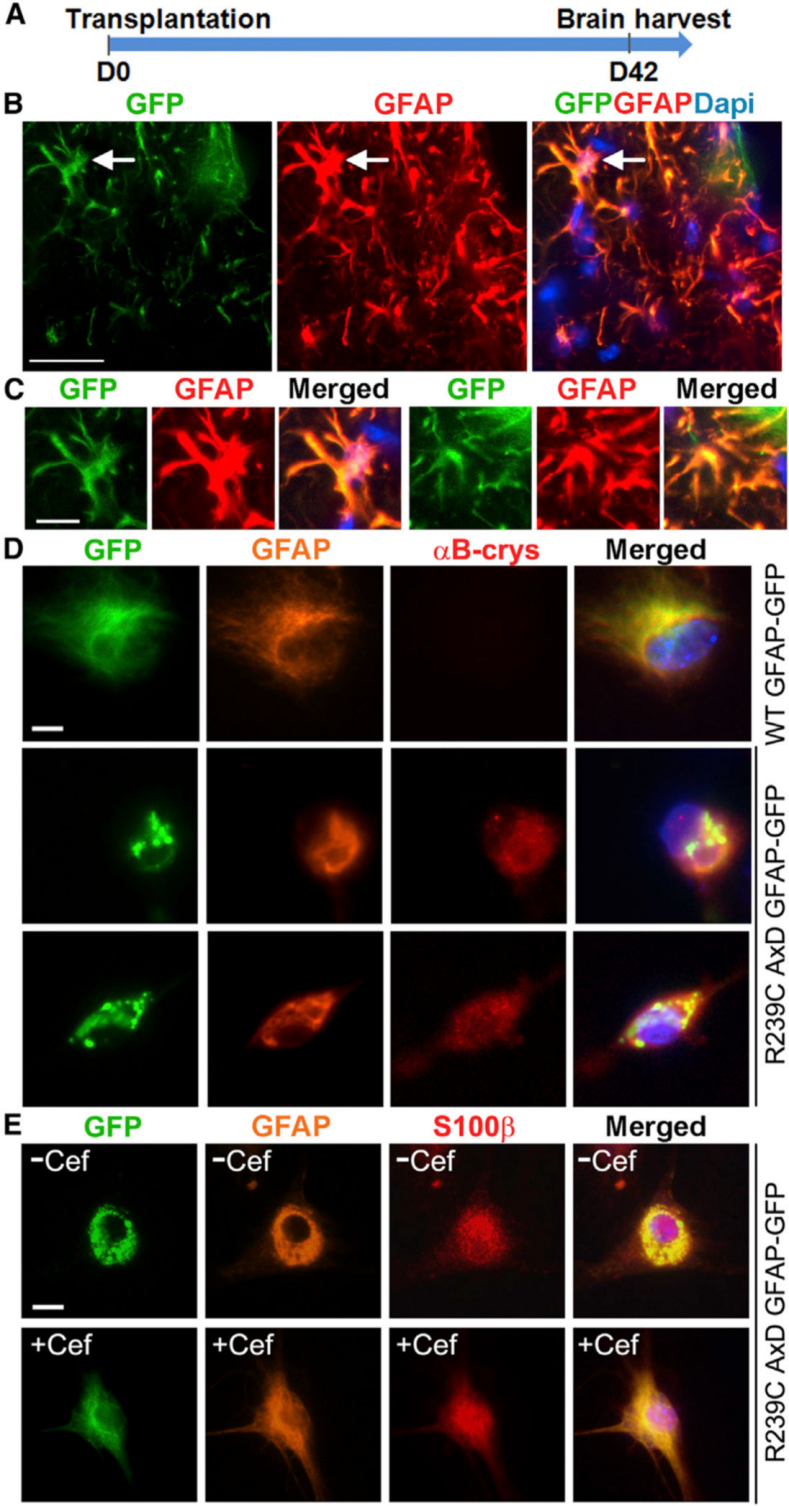
VCSTO-iA Can Survive Transplantation and Be Used for Disease Modeling (A) Timeline of cell transplantation and brain harvest. (B) GFP-labeled VCSTO- iAs in grafted brains that are positive for both GFP and GFAP. The endogenous astrocytes are shown as GFP negative but GFAP positive. The scale bar represents 25 μm. (C) Higher-magnification images of individual GFP and GFAP double-positive cells. One (left) represents the cell indicated by arrow in (B); another (right) represents a cell from a different region. The scale bar represents 10 μm. (D) Transfection of the R239C AxD mutant GFAP-GFP to iAs induced GFAP protein aggregates and αB-crystallin expression. iAs transfected with the WT and AxD GFAP-GFP were immunostained for GFAP and αB-crystallin. The transfected cells are indicated by GFP. Nuclei DAPI staining (blue) is included in the merged images. The scale bars represent 10 μm. (E) Ceftriaxone treatment reduced GFAP protein aggregates in iAs transfected with the AxD mutant GFAP-GFP. iAs transfected with the AxD GFAP-GFP were treated with ceftriaxone (+Cef) or vehicle control (−Cef) and stained for GFAP and S100β. The scale bar represents 10 μm. See also Figure S6.

To determine whether compound-iAs can survive and maintain their astrocytic identity in vivo, we labeled the VCSTO-iAs with a GFP reporter and transplanted them into the lateral ventricles of immunodeficient neonatal non-obese diabetic (NOD) severe combined immunodeficiency (SCID) gamma (NSG) mice (Figure 6A). Six weeks after transplantation, the grafted brains were analyzed by immunostaining. The GFP-positive grafted cells survived 6-week engraftment and continued to express GFAP in the brain (Figures 6B and 6C). In contrast, the control MEFs were not able to survive the engraftment in the transplanted brains (Figure S6A). These results indicate that the VCSTO-iAs can survive engraftment and maintain astrocytic marker expression in vivo.

### Compound-iAs Can Be Used to Model Neurological Disease

AxD is a neurological disease with astrocyte dysfunction and is caused by genetic mutation of the GFAP gene (Messing et al., 2012). Expression of AxD mutant GFAP induces the expression of αB-crystallin, a small heat shock protein, and the formation of protein aggregates containing GFAP and αB-crystallin in astrocytes (Messing et al., 2012). We tested whether compound-iAs could be used to model AxD. We transfected plasmid expressing GFP fusion of the wild-type (WT) or AxD mutant GFAP containing the R239C mutation, a hotspot mutation for AxD (Hagemann et al., 2006), into VCSTO-iAs. Expression of the AxD mutant GFAP in iAs promoted the expression of αB-crystallin and the formation of protein aggregates immunoreactive for GFAP and αB-crystallin, whereas transfection of the same amount of WT GFAP-GFP did not induce detectable αB-crystallin expression and GFAP protein aggregation (Figure 6D). The β-lactam antibiotic ceftriaxone has been shown to facilitate the elimination of AxD mutant GFAP protein aggregates in pAs (Bachetti et al., 2010). We treated VCSTO-iAs transfected with the AxD mutant GFAP with ceftriaxone. Substantial elimination of GFAP protein aggregates was detected in ceftriaxone-treated cells, compared to vehicle-control-treated cells (Figures 6E and S6B). Although MEFs transduced with the AxD mutant GFAP also exhibited GFAP protein aggregates, the fibroblast aggregates were not responsive to ceftriaxone treatment (Figure S6C).

**Figure 7.**
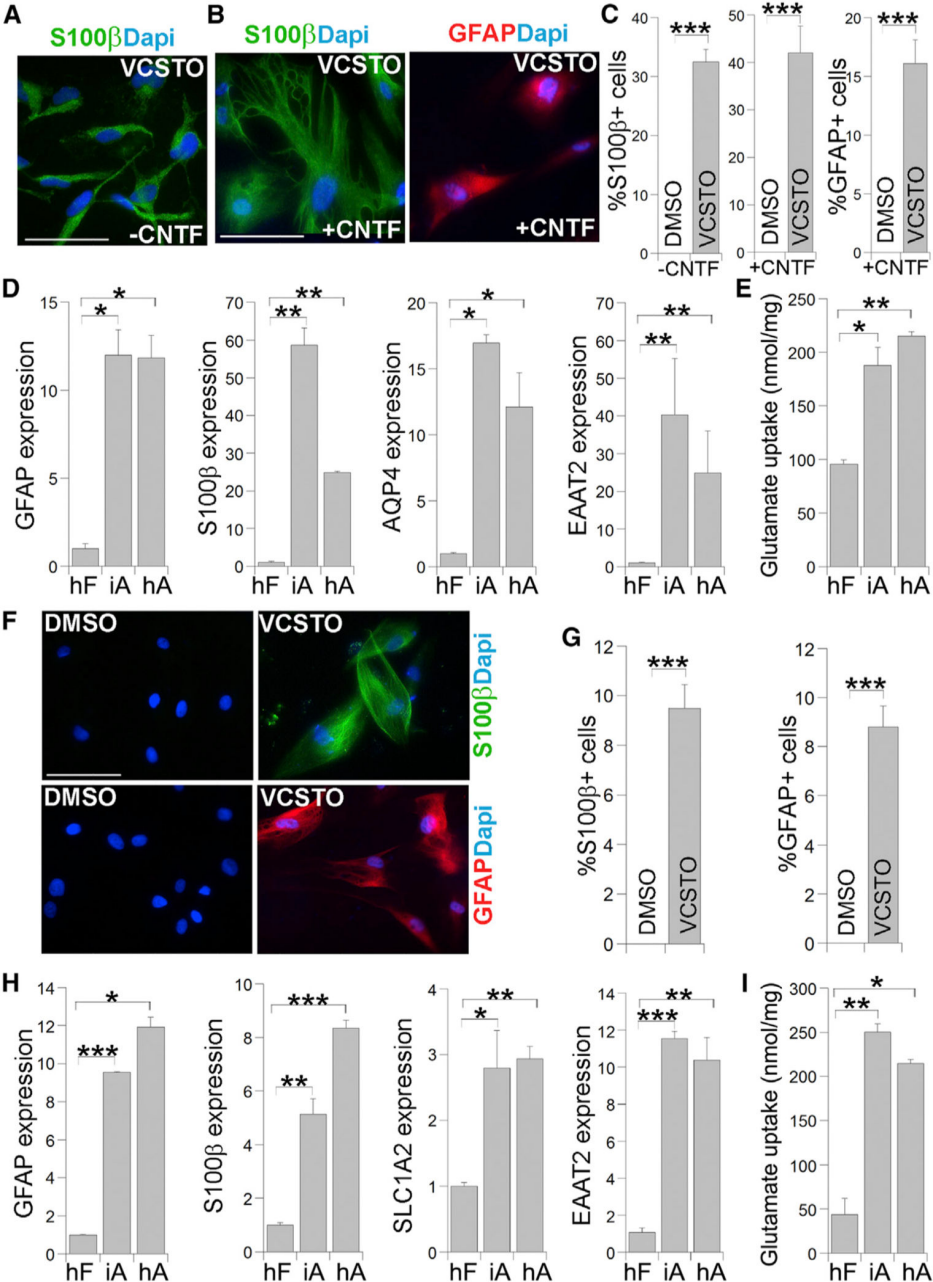
Reprogramming Human Fibroblasts into Astrocytic Cells by VCSTO (A) Immunostaining for S100β in cells derived from human fibroblasts (SCC058 for A–E) treated with DMSO or VCSTO. Nuclei were counter-stained with DAPI. The scale bar represents 100 μm for (A) and (B). (B) Immunostaining for S100β or GFAP in cells derived from human fibroblasts treated with DMSO or VCSTO, followed by CNTF treatment. (C) The percentage of S100b-positive cells or GFAP-positive cells is shown. n = 3,000–4,000 cells. (D) Expression of astrocyte marker genes in VCSTO-iAs as measured by real-time PCR. The expression in human fibroblasts (hFs) was defined as 1. n = 4 experimental repeats. (E) Substantially elevated glutamate uptake in iAsrelative to parental fibroblasts (hFs). n = 3 experimental repeats. (F) Immunostaining for S100β and GFAP in cells derived from human adult fibroblasts (AG14048 for F–I) treated with DMSO or VCSTO. Nuclei were counter-stained with DAPI. The scale bar represents 100 μm. (G) The percentage of S100β-positive cells or GFAP-positive cells is shown. n = 1,000 cells. (H) Expression of astrocyte marker genes in iAs relative to hFs as measured by real-time PCR. n = 4 experimental repeats. (I) Substantially elevated glutamate uptake in human iAs relative to hFs. n = 3 experimental repeats. See also Figure S7.

S100β is a marker for human astroglial progenitor cells and astrocytes, we stained the VCSTO-treated cells for S100β and found that more than 30% of cells were S100β+ cells, whereas no S100β+ cells were detected in DMSO-treated cells (Figures 7A and 7C). Treatment of the astroglial progenitor cells with ciliary neurotrophic factor (CNTF) for 6 days allowed the maturation of these

Moreover, glutamate uptake assay revealed that VCSTO-iAs transduced with the AxD mutant GFAP exhibited reduced glutamate uptake, compared to VCSTO-iAs transduced with WT GFAP (Figure S6D). These results together suggest that compound-iAs could be used to model neurological diseases with astrocyte dysfunction and test candidate drugs for these diseases.

### VCSTO Could Induce Astrocytic Conversion from Human Fibroblasts

To determine whether human fibroblasts could be induced for astrocytic conversion using small-molecule compounds, we treated human foreskin fibroblasts with VCSTO compounds. Forty days after compound treatment, we observed a large number of cells with astroglial progenitor-like morphology. Because cells into astrocytes with bigger cell body and more-complex morphology. Immunostaining of the resultant cells allowed the detection of both S100β+ cells and GFAP+ cells in VCSTO-treated cells (Figures 7B and 7C).

In a parallel experiment, we detected robust induction of astrocyte marker genes, GFAP, S100b, AQP4, and EAAT2, in VCSTO-reprogrammed cells (iAs), to a level that is similar to or higher than that in human iPSC-derived astrocytes (hA) (Figure 7D). In contrast, the expression level of the astrocytic genes is much lower in DMSO-treated human fibroblasts (hF) (Figure 7D). Moreover, human iAs exhibited potent glutamate uptake, compared to parental fibroblasts (Figure 7E). Calcium imaging analysis revealed that the human iAs exhibited glutamate-induced calcium signal change, similar to hA, whereas parental fibroblasts did not exhibit calcium signal change in response to glutamate stimulation (Figure S7). These results together indicate that the VCSTO cocktail could induce human fibroblasts into astroglial progenitor cells that can be further matured into functional astrocytes.

To determine whether adult human fibroblasts could be induced into astrocytes using small molecules, we treated human fibroblasts derived from a 71-year-old donor with the VCSTO compounds. After 40 days of VCSTO compound treatment and 10 days of CNTF-induced maturation, both S100β+ cells and GFAP+ cells were detected in VCSTO-treated cells, but not in DMSO-treated cells (Figures 7F and 7G). RT-PCR analysis revealed potent induction of astrocyte marker genes, GFAP, S100β, SLC1A2, and EAAT2 in VCSTO-induced cells, compared to that in DMSO-treated cells (Figure 7H). The iAs also exhibited substantial glutamate uptake, compared to parental fibroblasts (Figure 7I). These results together indicate that the VCSTO compounds could induce human adult fibroblasts into functional astrocytes.

## DISCUSSION

In this study, we reprogrammed mouse fibroblasts into functional astrocytes, which possess the ability to promote neuronal maturation and synaptic formation, uptake glutamate, and induce calcium signal in response to glutamate stimulation. Although rapid progress has been made in converting somatic cells into other types of neural cells, such as neural stem cells, neurons, and oligodendrocytes, direct reprogramming of somatic cells into astrocytes remains largely behind. Induced neurons can be developed into useful tools for modeling a variety of neurological diseases affecting neurons (Lujan et al., 2012). However, induced neuronal cells would have limitations for modeling disease affecting astrocytes. Although astrocytes could be derived from iPSCs, the differentiation process is lengthy. These limitations could be overcome by inducing astrocytes directly from fibroblasts in a relatively short period of time as reported in this study.

In this study, we present an example of pure chemical induction of lineage conversion from a mature somatic cell type to astrocytes. A study reported the derivation of iPSCs from mouse somatic cells using six small molecules VC6TFZ (Hou et al., 2013). Part of this compound cocktail, VC6 was used to convert somatic cells into neural progenitor cells under hypoxia (Cheng et al., 2014). In recent studies, different combinations of small molecules were used to convert somatic cells into neuronal cells (Hu et al., 2015; Li et al., 2015; Zhang et al., 2015). In this study, we directly reprogrammed mammalian fibroblasts into astrocytes using compounds only, without using any transgenes or viral transduction. Moreover, we found that the TGFβ inhibitor 6 or S alone was able to induce GFAP-positive cells from MEFs, providing an example that one single compound is able to induce the conversion of one somatic cell type to another.

Several possible reasons could explain why inhibition of TGFβ pathway could induce reprogramming of fibroblasts into astrocytes. First, TGFβ is a cytokine for induction of epithelial-to-mesenchymal transition. Inhibition of TGFβ signalling could induce reprogramming of fibroblasts by suppressing the fibroblast gene expression program through a mesenchymal-to-epithelial conversion (Lin et al., 2009; Maherali and Hochedlinger, 2009). Second, inhibition of TGFβ signaling has been shown to promote neuroectoderm specification (Smith et al., 2008). Moreover, inhibition of TGFβ signaling can induce bone morphogenetic protein (BMP) signaling (Xu et al., 2008; Ichida et al., 2009), which has been shown to induce astrocytic differentiation and establish and maintain astrocytic identity (Gross etal., 1996;RajanandMcKay,1998; Bonaguidi et al., 2005; Kohyama et al., 2010).

Previous studies described ways to derive astrocytes from somatic cells by going through iPSC or induced neural stem cell (iNSC)/induced neural progenitor cell (iNPC) intermediates (Han et al., 2012; Lujan et al., 2012; Ring et al., 2012; Thier et al., 2012; Cassady et al., 2014). In these studies, astrocytes were derived from MEFs at an efficiency of 0.004%–2% in up to 70 days. Our method of direct reprogramming does not go through iPSC or iNSC/iNPC intermediate state. Astrocytes could be converted from MEFs at an efficiency of 38% in 20–25 days in this study. Therefore, the direct chemical reprogramming method described in this study provides a more rapid and efficient way to derive astrocytes from fibroblasts.

Furthermore, this study provides proof of concept that chemical iAs can be used to model diseases with astrocyte dysfunction. When we transfected an AxD mutant GFAP into iAs, we were able to recapitulate the phenotype of GFAP protein aggregation observed in AxD patient astrocytes. Moreover, these protein aggregates were responsive to ceftriaxone treatment. Our knowledge about astrocytes is still very limited. The chemical iAs developed in this study will provide a tool for us to study neurodevelopment in glial context and to model a variety of neurological diseases with astrocyte dysfunction. Generating iAs containing disease-causing mutations will provide novel insights into our understanding of astrocyte-associated diseases.

## EXPERIMENTAL PROCEDURES

### Cell Culture

MEFs and TTFs were derived from embryonic day 13.5 (E13.5) embryos of Oct4-GFP transgenic (OG2; Szabó et al., 2002; kindly provided by Dr. Szabo), WT, or GFAP-GFP transgenic mice (Jackson Laboratory; Zhuo et al., 1997). These cells were cultured in MEF medium containing DMEM, 10% fetal bovine serum (FBS), 0.1 mM nonessential amino acids, and 2 mM L-glutamine. Mouse pAs were isolated from postnatal day 1 (P1)–2 pups following published protocol (Schildge et al., 2013) and cultured in DMEM containing 10% FBS.

### Direct Reprogramming Mouse Fibroblasts into Astrocytes

MEFs were plated on 6-well or 12-well plates at a cell density of 3 × 10^3^cells/cm^2^. Cells were cultured in MEF medium for 24 hr and then changed to mouse iAM containing knockout DMEM with 10% knockout serum replacer, 10% FBS, 2mML-glutamine, 0.1mM non-essential amino acids (NEAA), 0.1mM β-mercaptoethanol, and 100 ng/ml fibroblast growth factor (FGF). Cells were treated with compounds, including 500 nM valproic acid (VPA) (Stemgent), 3 μM CHIR99021 (D&C Chemicals), 10 mM SB-431542 (D&C Chemicals), 10 μM tranylcypromine (Stemgent), and 1 μM OAC1 (Lietal.,2012) for 10days; re-plated onto Matrigel-coated plates (BD Biosciences); and continued with compound treatment for another 15 days. Cells were then switched to mouse astrocyte medium (AM) containing DMEM with 10% heat-inactivated FBS.

### Immunocytochemistry

Cells were fixed in 4% paraformaldehyde (PFA) for 10 min, followed by washes in PBS at room temperature (RT). Cells were then blocked with 3% donkey serum in PBS containing 0.01% Triton X-100 for 1 hr at RT, incubated with primary antibodies overnight at 4°C, and then washed with PBS and incubated with secondary antibodies for 1 hr at RT. We used primary antibodies for GFAP (1:2,000; DAKO; Z0334), S100β (1:200; NOVUS; NB110–57478), ALDH1L1 (1:200; NeuroMab; 75–140), synapsin (1:1,000; SYSY; 106103), αB-crystallin (1:200; Enzo; ADI-SPA-223), MAP2 (1:500; GeneTex; GTX11268), Tuj1 (1:6,000; Covance; PRB-435P), NeuN (1:400; Millipore; MAB377), Pax6 (1:500; Covance; PRB-278P), Sox1 (1:500; Millipore; AB15766), Oligo2 (1:200; GeneTex; GTX62440), NG2 (1:500; Millipore; MAB5384), and NKX2.2 (1:50; DSHB; 745A5). Nuclei were stained with DAPI (1:6,000; Sigma; D9564).

### Cell Sorting and Microarray Gene Expression Analysis

GFAP-GFP-positive iAs were sorted using the FACSAria III cell sorter (BD Bioscience). Gene expression profiling was performed using Mouse Gene 2.0 ST array (Affymetrix). Microarray data analysis was performed using Partek Genomics Suite (Partek). Expression values were robust multi-array average (RMA) normalized (Irizarry et al., 2003). Fold-change values represent the linear ratio between signal intensities when the ratio value is greater than 1 and −1/ ratio when the ratio is less than 1. Genes were defined as differentially expressed if they showed a fold-change value >1.5. Heatmaps to visualize differentially expressed genes were produced in Partek using Euclidian distance for hierarchical clustering of standardized expression values. GO enrichment was performed for functional enrichment of commonly affected genes, with p values calculated via Fisher’s exact test.

### Real-Time PCR

Total RNA was extracted using Trizol reagent (QIAGEN); cDNAs were prepared using Tetro cDNA synthesis kit (Bioline). Real-time PCR was performed using DyNAmo Flash SYBR Green qPCR mix on a StepOnePlus system (Applied Biosciences) and normalized to β-actin. Primers used are listed in Table S1.

### Astrocyte-Neuron Co-cultures

Mouse cortical neurons were isolated from E13.5 mouse embryo and cultured in neuronal culture media (neurobasal; 1X B27; 2 mM L-glutamine) alone or directly on a layer of iAs, mouse pAs, or MEF for 5 days. Mouse neurons, iA, pAs, or MEFs were plated at the same density of 10,000 cells/cm^2^. The cocultured cells were stained for MAP2 and synapsin. The synapsin + puncta along the MAP2+ neurites were expressed as the number of puncta per 50-μm neurite length.

### Transplantation

iAs labeled by GFP-expressing lentivirus were dissociated using trypsin-EDTA and resuspended in medium at 100,000 cells/ml density and kept on ice. Two-microliter cell suspensions were injected 1 mm from the midline between the Bregma and Lambda and 1 mm deep into the anterior lateral ventricles of immunodeficient neonatal NSG mice. After 6 weeks, mice were euthanized and perfused with 4% PFA for 5 min. Brain tissues were harvested for immunostaining. All animal work was performed under the Institutional Animal Care and Use Committee (IACUC) protocol approved by the City of Hope IACUC Committee.

### Ca^2+^ Imaging

Cells were seeded in 12-well plates at a density of 1 × 10^5^ cells per well and stimulated with 10 μM glutamic acid. Fluo-4 Calcium Imaging Kit (Invitrogen F10489) was used to monitor calcium waves following manufacturer’s instructions. Calcium waves were captured using a Zeiss Observer Microscope. Wave intensity was analyzed using Image Pro Premier, and the intensity was measured as ΔF/F0 = (F -F0)/F0.

### Bisulfite Sequencing

Genomic DNAs were isolated from MEF, fluorescence-activated cell sorting (FACS)-sorted iAs, and mouse pAs using a Genomic DNA Purification Kit (QIAGEN). Bisulfite conversion of genomic DNAs was carried out using the EZ DNA Methylation-Gold Kit (Zymo Research). The bisulfite-modified DNA was then used as a template for PCR to amplify the promoter region of Gfap. The amplified products were cloned into the pCR2.1-TOPO cloning vector (Invitrogen), and ten randomly selected clones were sequenced using T7 or M13R primers.

### Glutamate Uptake Assay

The glutamate uptake was measured using the Glutamate Assay Kit (BioVision). iAs were plated at a concentration of 2 × 10^4^ cells per well in a 24-well plate. One hundred micromolar L-glutamate was added to each well. After incubation for 6 hr, the glutamate concentration in the media was measured and presented as nmol of glutamate per mg of total proteins.

### Transfection into iAs

iAs were seeded at 1 × 10^5^ cells per well in 12-well plates and incubated overnight. Then, 2 μg plasmid of human WT GFAP-GFP or AxD mutant GFAP-GFP with the R239C mutation (Bachetti et al., 2008) was transfected into iAs using Lipofectamine 2000 (Invitrogen). Forty-eight hours after transfection, cells were assayed by immunostaining. For drug treatment, 24 hr after transfection, cells were treated with vehicle control or 100 μM ceftriaxone for 48 hr, followed by immunostaining.

### Inducing Human Fibroblasts for Astrocytic Conversion

Human fibroblasts were purchased from Millipore (SCC058) or Coriell (AG14048) and tested for lack of mycoplasm contamination. Human fibro-blasts were seeded onto 6-well plates at the density of 10^4^ cells/cm^2^ and cultured in either Fibro-GRO complete medium (for SCC058) or Eagle’s minimum essential medium (MEM) with 15% non-inactivated FBS (for AG14048) for 24 hr and then switched to iAM containing DMEM/F12 with 2 mM L-glutamine, 0.1 mM NEAA, 1×N2, 1×B27, and 100 ng/ml FGF. For SCC058, cells were treated with VCSTO compounds, including 500 nM VPA (Stemgent), 3 μM CHIR99021 (D&C Chemicals), 10 μM SB-431542 (D&C Chemicals), 10 μM tranylcypromine (Stemgent), and 1 μM OAC1 (Li et al., 2012) for 20 days, re-plated onto Matrigel-coated plates, and continued with compound treatment for another 20 days. Cells were then treated with 10 ng/ml CNTF for another 6 days. For AG14048, cells were treated with VCSTO compounds at the same concentration as described above for 30 days and then treated with VCSTO together with 10 ng/ml CNTF for another 10 days.

### Statistical Analysis

Independent-samples t test was used to compare means of two independent samples. A value of p < 0.05 was considered statistically significant.

## Supplementary Material

Supplemental material

## Highlights

- Fibroblasts can be directly converted into astrocytes with a chemical cocktail
- Induced astrocytes resemble primary astrocytes in gene expression profile
- Induced astrocytes exhibit similar functional properties as primary astrocytes
- Induced astrocytes can be used for disease modeling and drug discovery
