# Supplementary material for: Small-Molecule-Based Lineage Reprogramming Creates Functional Astrocytes
Source: Cell Rep. Author manuscript; Available in PMC 2022 Jun 24. (PMC9228989; doi:10.1016/j.celrep.2016.06.042)
Supplement: Supplemental material [file NIHMS1801534-supplement-Supplemental_material.pdf]

**Cell Reports, Volume *16***

# Supplemental Information

## Small-Molecule-Based Lineage Reprogramming Creates Functional Astrocytes

**E Tian, Guoqiang Sun, Guihua Sun, Jianfei Chao, Peng Ye, Charles Warden, Arthur D. Riggs, and Yanhong Shi**

**SUPPLEMENTAL FIGURES AND LEGENDS**

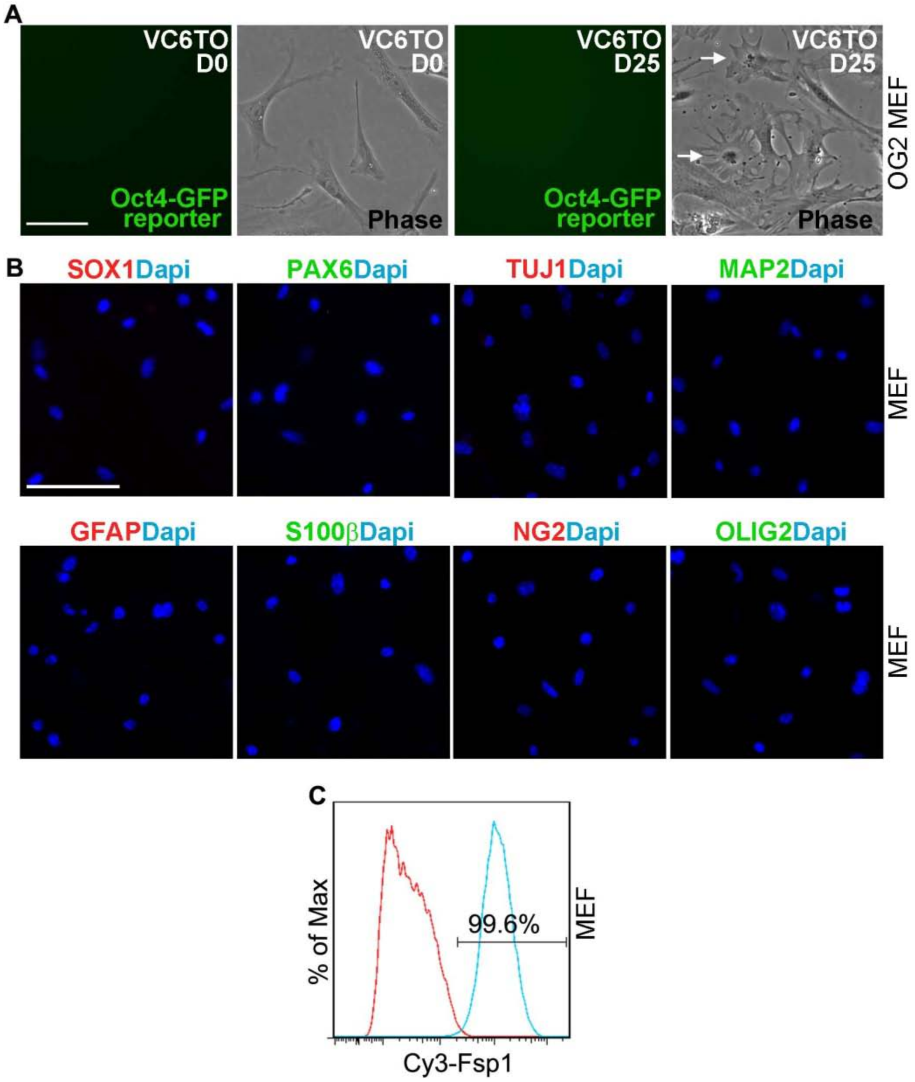


**Figure S1 Direct reprogramming MEF into astrocyte-like cells, Related to Figure 1.**
(A) VC6TO induced OG2 MEF into cells with astrocyte-like morphology. Oct4 promoter driven-GFP reporter fluorescence and phase contrast images of OG2 MEF before (D0) and after 25 days of VC6TO treatment (D25). No induction of Oct4-GFP reporter was detected. Arrows indicate astrocyte-like cells. Scale bar: 100 µm. (B) No contamination of neural lineage cells in MEF preparation. MEFs were stained for neural progenitor markers SOX1 and PAX6, neuronal markers TUJ1 and MAP2, astrocytes markers GFAP and S100β, and oligodendrocyte progenitor cell markers NG2 and OLIG2. No staining with these markers was detected. Nuclei Dapi staining is shown in blue. Scale bar, 100 µm. (C) FACS analysis showing 99.6% Fsp1+ cells in MEF.

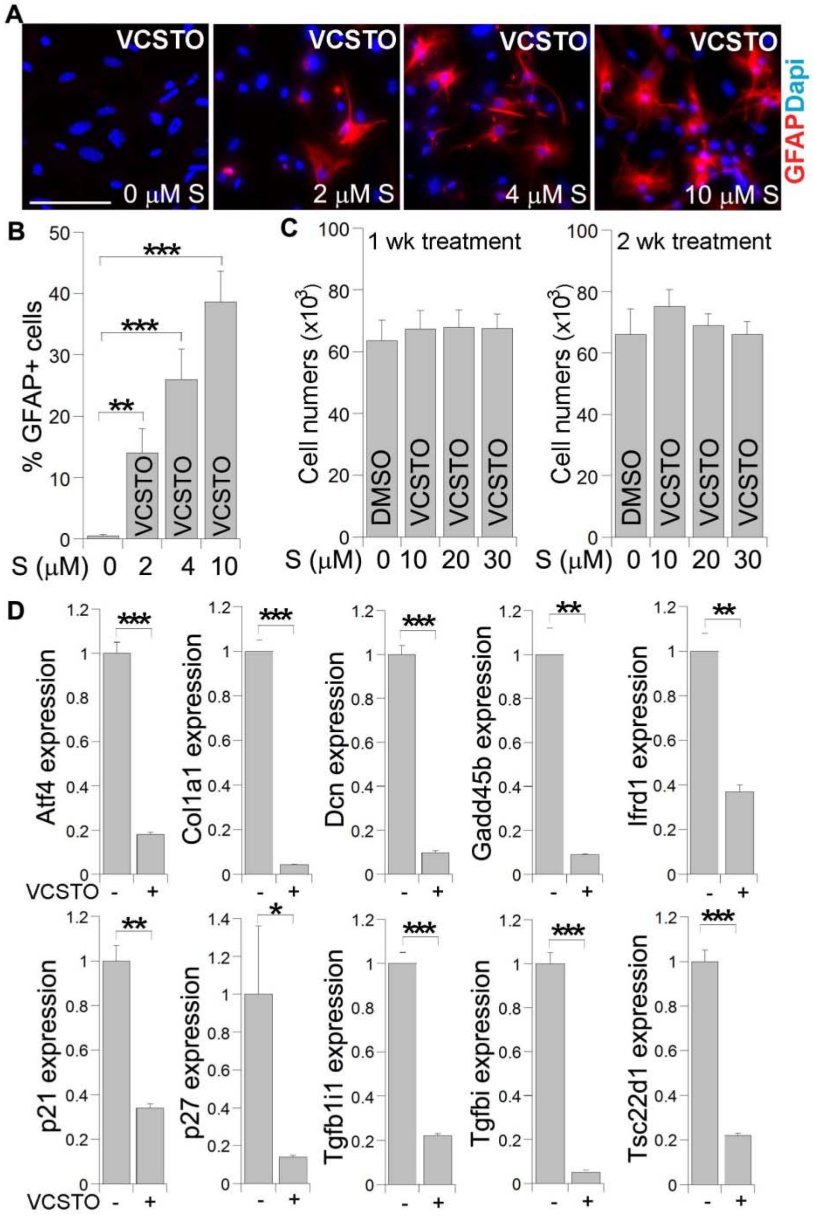


**Figure S2 Treatment with VCSTO inhibited TGFβ target genes, Related to Figure 2.** (A) Immunostaining for GFAP in cells reprogrammed from MEFs treated with VCSTO at different concentrations of S (SB-431542). Scale bar, 100 μm. (B) The percentage of GFAP-positive cells in MEFs treated with VCSTO at different concentrations of S. n=2,000 to 6,000 cells counted for each treatment group. (C) Treatment with VCSTO exerted no cellular toxicity. MEFs were treated with DMSO or VCSTO with increasing concentrations of S. Cell toxicity was determined by counting live cells 1 week or 2 weeks after vehicle or compound treatment. Error bars are sd of the mean. n=4 experimental repeats for each treatment group. (D) Relative expression levels of TGFβ downstream target genes in MEFs treated with vehicle only or VCSTO compounds, measured by real time PCR. The expression in MEFs treated with vehicle control was defined as 1. n=3 experimental repeats.

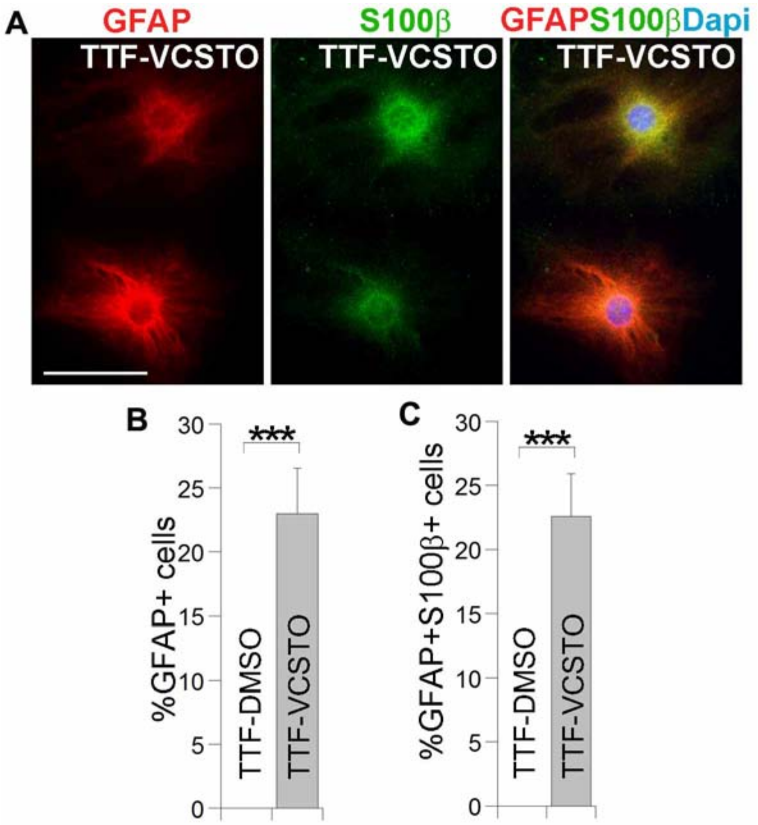


**Figure S3 Astrocytic reprogramming by VCSTO in tail tip fibroblasts, Related to Figure 2.** (A) Immunostaining for GFAP and S100β in cells generated from tail tip fibroblasts (TTFs) treated with VCSTO for 25 days. Nuclei Dapi staining is shown in blue in the merged image. Scale bar, 50 µm. (B, C) Quantification of the percentage of GFAP-positive cells (B) and GFAP and S100β-double positive cells (C) in total Dapi-positive cells. For panels B and C, error bars are sd of the mean. ***p<0.001. n=967 cells for TTF-DMSO, n=1118 cells for TTF-VCSTO.

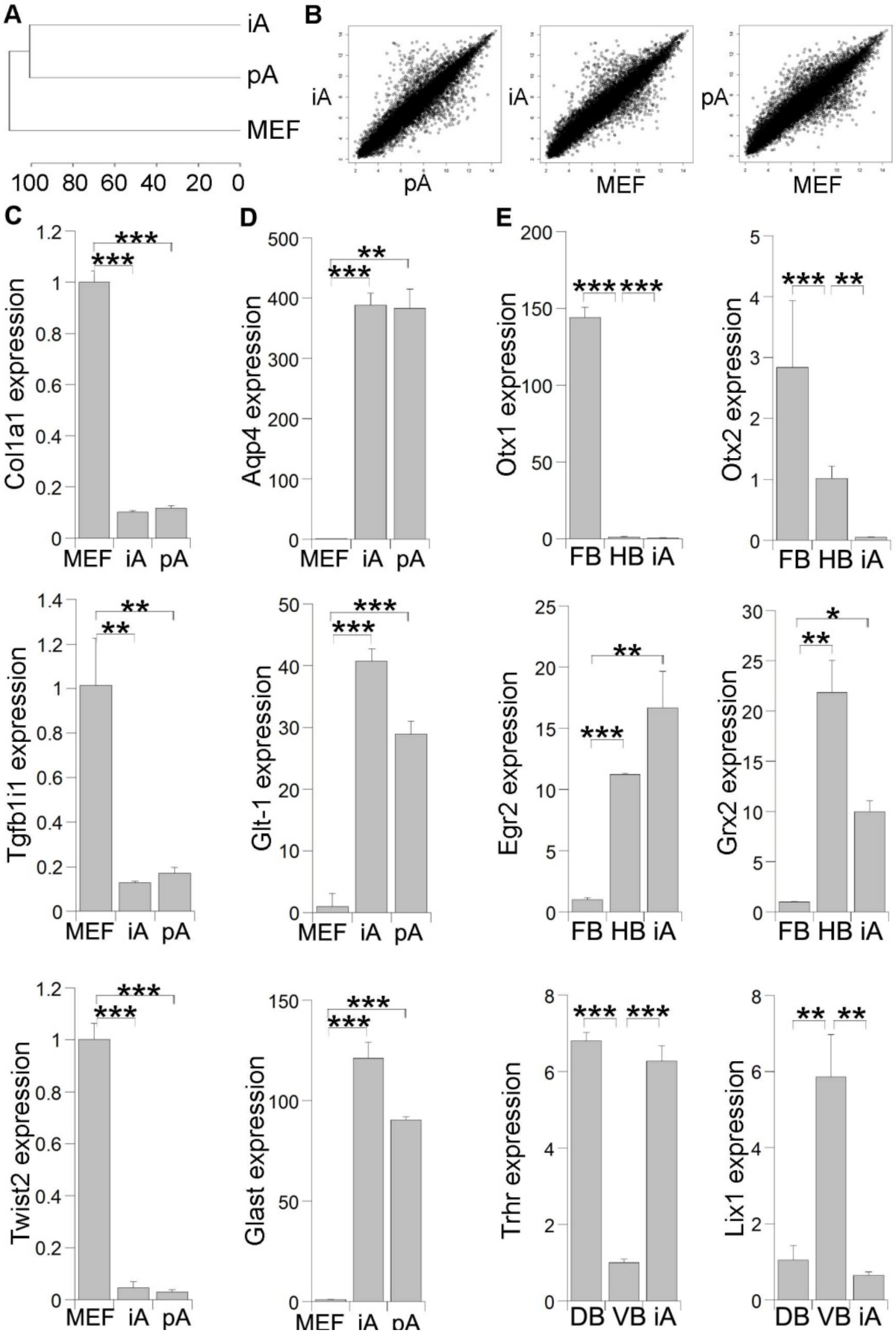


**Figure S4 Gene expression analysis of VCSTO-induced astrocytes, Related to Figure 4.** (A) Hierarchical clustering of induced astrocytes (iA), primary astrocytes (pA) and MEF based on all the probe sets in microarray data. (B) Pearson's correlation analysis of iA, pA, and MEF. (C, D) RT-PCR analysis of 3 fibroblast genes (C) and 3 astrocyte marker genes (D) in MEF, iA and pA. The expression in MEF was defined as 1. (E) Relative expression of regional subtype markers in iA measured by real time PCR. FB: forebrain; HB: hindbrain; DB: dorsal brain; VB: ventral brain. Error bars are sd of the mean. $^{**}p<0.01$, $^{***}p<0.001$ by Student's t-test. n=3 experimental repeats.

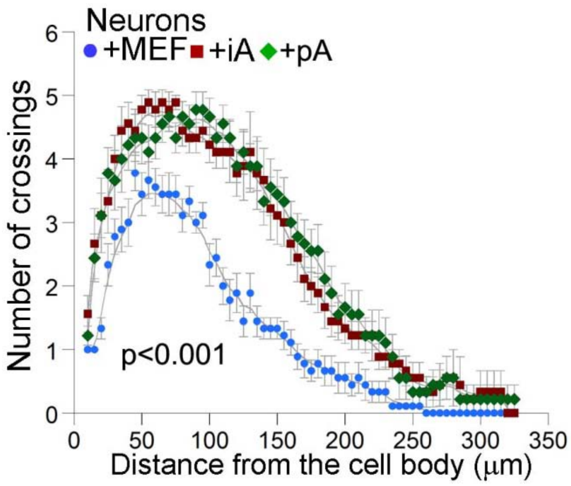


**Figure S5 Sholl analysis of neurite complexity in co-cultured neurons, Related to Figure 5.** Neurons were co-cultured with MEF, induced astrocytes (iA), or mouse primary astrocytes (pA), respectively. Errors bars are se of the mean.

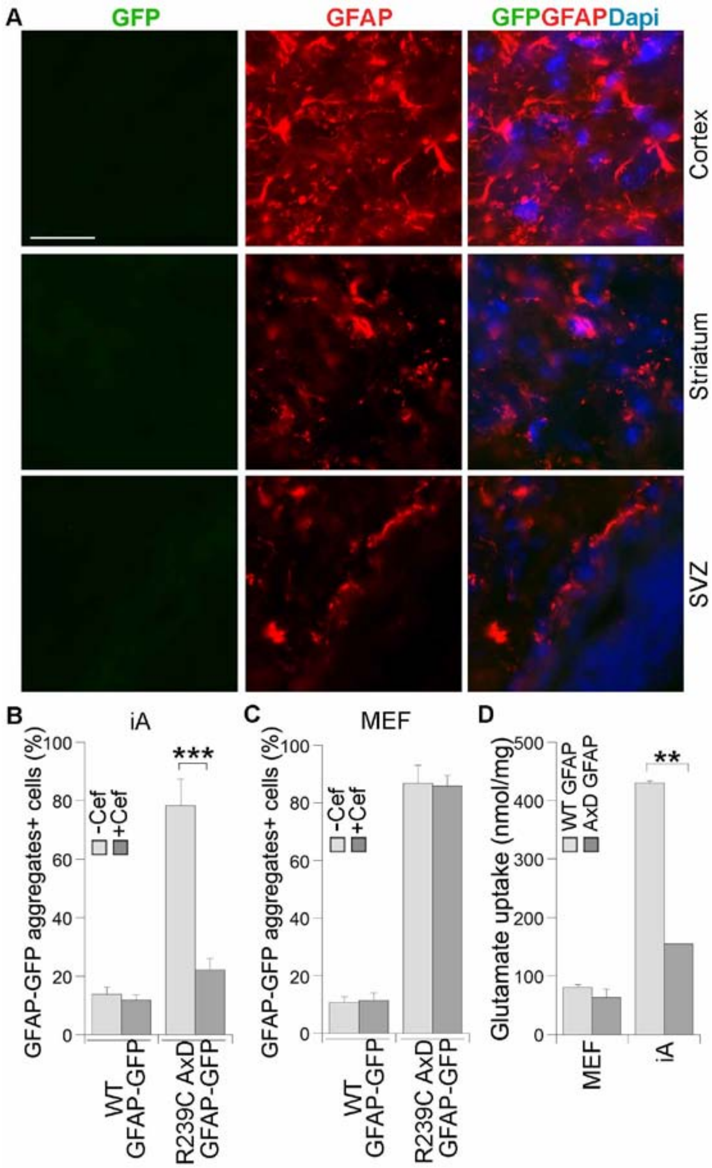


**Figure S6 Induced astrocytes in disease modeling, Related to Figure 6.**
(A) MEF failed to survive in transplanted brains. MEF were labeled by GFP and transplanted into brains of neonatal immunodeficient mice. No GFP signal was detected in various regions of MEF-transplanted brains two weeks after transplantation. Scale bar, 25 µm. (B) Ceftriaxone treatment reduced GFAP protein aggregates in induced astrocytes (iA) transfected with the AxD mutant GFAP-GFP. The percentage of iA that contain the GFAP-GFP aggregates is reduced upon ceftriaxone treatment. n=400 to 500 cells. (C) Ceftriaxone treatment had no effect on GFAP protein aggregates in MEF. MEF transduced with the AxD GFAP-GFP were treated with ceftriaxone (+Cef) or vehicle control (-Cef). The percentage of MEF that contain the GFAP-GFP aggregates remained the same in the absence or presence of ceftriaxone treatment. n=600 to 900 cells. (D) Reduced glutamate uptakte in iA tranduced with AxD mutant GFAP, compared to iA transduced with WT GFAP. n=3 experimental repeats. Error bars are sd of the mean, **$p<0.01$ by Student's t-test.

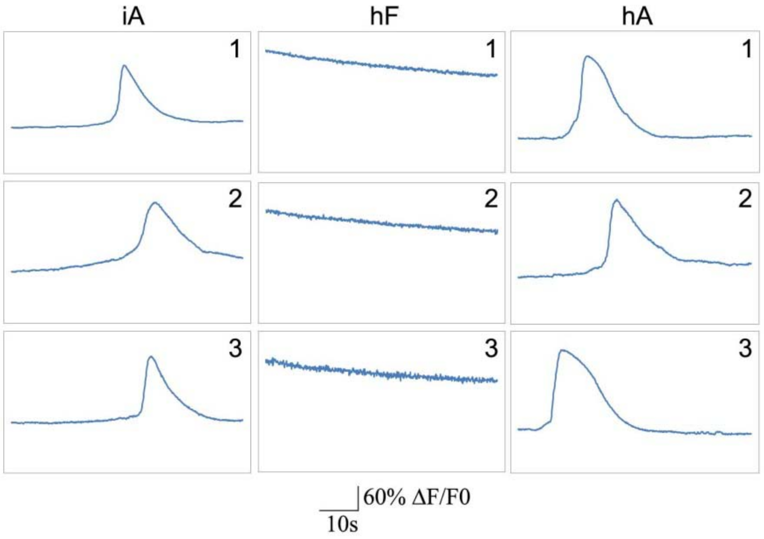


**Figure S7 Calcium imaging analysis of human induced astrocytes, Related to Figure 7.** Human induced astrocytes (iA) exhibited calcium signal change in response to glutamate stimulation, similar to human iPSC-derived astrocytes (hA). Lack of calcium spikes after glutamate stimulation in human fibroblasts (hF) was included as a negative control.

## SUPPLEMENTAL EXPERIMENTAL PROCEDURES

### FSP1 FACS analysis

MEFs were stained by FSP1 antibody (Millipore, ABF32). FACS analysis was performed on Flow Cytometry Analyzers BD Fortessa (BD Bioscience).

**Table S1. Primers used for RT-PCR.**

| Gene | Forward sequence | Reverse sequence |
|---|---|---|
| *Gfap* | GAAGTTCGAGAACTCCGGGAG | TTAGACCGATACCACTCCTCTG |
| *mS100B* | CCCTCATTGATGTCTTCCACCA | CTTCGTCCAGCGTCTCCATCAC |
| *Aldh1L1* | GGAAGACAGCAGCCTGCCTG | CTTCACATTACTCAGGGCAC |
| *NFIA* | ACAGGTGGGGTTCCTCAATC | GAGGCTTGGTGTCTGGCATG |
| *Ctnnal1* | GGAGAAGGTCACGGAGATCG | TGAAGTCCTCCACATGCTCC |
| *Ndp* | TGGACTCTCAACGCTGCATG | GGACAGTGCTGAAGGACACC |
| *Lix1* | GGAGTTCATCATGGAGAGTG | CATTCCAGTGCAGTAGTTGG |
| *Sox6* | CTTGCCGATGTGGTGGATAC | CTCTGCAAGGCTCTCAGGTG |
| *Tlr3* | TCACTTGCTCATTCTCCCTT | GACCTCTCCATTCCTGGC |
| *Let7b* | TCGACTCGAGCCCTCTCACTGAACCTCTGTCTCC | GCGAATTCTTAATTAAAAACCACCCAATCTGTGGCTCCA |
| *Col12a1* | AGGAGTTGATGAGCAGCTTG | GGTCACGAACATTGAGCGTG |
| *Col6a3* | TCTTGAACGTGTGGCTAACC | TCTCCAGAGCACTTGCATGG |
| *Col3a1* | GCCCACAGCCTTCTACAC | CCAGGGTCACCATTTCTC |
| *Col1a1* | GCAACAGTCGCTTCACCTACA | CAATGTCCAAGGGAGCCACAT |
| *P21* | AATCCTGGTGATGTCCGACC | CAAAGTTCCACCGTTCTCGG |
| *ATF4* | GAGCTTCCTGAACAGCGAAGTG | TGGCCACCTCCAGATAGTCATC |
| *P27* | GCCTGACTCGTCAGACAATC | CGTCTGCTCCACAGTGCCAG |
| *DCN* | TGAGCTTCAACAGCATCACC | AAGTCATTTTGCCCAACTGC |
| *Gadd45b* | CCTGGCCATAGACGAAGAAG | AGCCTCTGCATGCCTGATAC |
| *IFRD1* | ACAGGCAGCTCTTGAAGGTC | AGACAGCGCTCAATGCTATC |
| *TGFB1I1* | GCCTCTGTGGCTCCTGCAATAAAC | CTTCTCGAAGAAGCTGCTGCCTC |
| *TGFBI* | CCAAGTCACCCTACCAGCTG | TCCTCTGGTACCACTGCTTG |
| *TSC22D1* | GCTGCTGCTGCTGTCTGAAC | ACATCCCTGCTCACTCTCTG |
| *FOXG1* | TGGCAACACTGCCCATTCA | GCATTTGCGCAACACAGGTTA |
| *Hoxb4* | TTCACGTGAGCACGGTAAAC | CACTTCATGCGCCGATTCTG |
| *NKX2.1* | AAAACTGCGGGGATCTGAG | TGCTTTGGACTCATCGACAT |
| *Pax3* | ACTACCCAGACATTTACACCAGG | AATGAGATGGTTGAAAGCCATCAG |
| *Sox1* | CCAAGAGACTGCGCGCGCTG | TGAGCAGCGTCTTGGTCTTG |
| *Pax6* | ACCAAAGGGTCATCGCGCCC | TGGCAGTCCTTGCGATCGGC |
| *Oct4* | TAGGTGAGCCGTCTTTCCAC | GCTTAGCCAGGTTCGAGGAT |
| *Nanog* | CAGGAGTTTGAGGGTAGCTC | CGGTTCATCATGGTACAGTC |
| *AQP4* | TTCTCTTCGGTGCTAGGAAAC | AGGAAGCTTATGTCTCTGGTG |
| *Glast1* | CTAGTTGTCTTCTCCATGTG | AGGAGAGGCAGGACGATGAC |
| *Glt1* | GCGCATGTGCGACAAGCTGG | GCGATGCCAAGCGAAGCAGC |
| *36B4* | TGGTGCTGATGGGCAAGAA | ATTCCCCCGGATATGAGGC |
| *Actin* | CCGAGCGTGGCTACAGCTTC | ACCTGGCCGTCAGGCAGCTC |
| m*Gfap* | TTGTTGGTATGGAGTATAGGTTGTTGTTAT | CCTACCTTCCTCTACCCATACTTAAACT |
